# Dataset of average P-wave velocities of lithostratigraphic units in the middle and southern Adriatic Sea (Italy): Extrapolation from digitalized sonic logs of exploration wells within ViDEPI project

**DOI:** 10.1016/j.dib.2026.113035

**Published:** 2026-06-28

**Authors:** C. Lombardo, M. Cicala, F. De Giosa, L. Sabato, G. Scicchitano, L. Spalluto, G. Tagliaro, M. Tropeano, V. Festa

**Affiliations:** a Dipartimento di Scienze della Terra e Geoambientali, Università degli Studi di Bari Aldo Moro, Via E. Orabona 4, 70125 Bari, Italy; b Environmental Surveys S.r.l. (ENSU), Spin-Off Università degli Studi di Bari Aldo Moro, Via D. Lupo 65, 74121, Taranto, Italy; c Oceanographic Institute, University of São Paulo, Praça Oceanográfico 191 - Vila Universitaria, 05508-120, São Paulo, Brazil

**Keywords:** Seismic velocity, Log ASCII Standard (LAS), Time–depth conversion, Well logs, Petrel™

## Abstract

The present data article deals with a dataset concerning sonic log–derived P-wave average velocities. Twelve offshore exploration wells (drilled between 1969 and 2000) in the middle/southern Adriatic Sea (pertaining Italy), have been taken into account. The used data consisted in converted legacy analog well-log information into standardized digital formats for their reuse in geological and geophysical applications. In particular, original raster well-log documents from the public Visibility of Data related to Oil Exploration Activities in Italy database were digitized to obtain sonic log data in CSV format, which consisted of interval transit time (Δt) values and their corresponding depths (referenced to sea level and accounting for rotary table height).

Data processing to achieve the sonic log–derived P-wave average velocities was performed using Petrel™ software. A consistent, multi-step workflow was applied to all wells. In the first step, lithostratigraphic units were identified on the basis of the raster stratigraphic logs and defined within the software, by entering unit names, depths, and graphical attributes. Next, sonic log data were imported/converted in TXT and grouped according to the lithostratigraphic units. A block-averaging procedure was applied to each unit to reduce local variability in Δt values; therefore, representative average interval transit times (Δt_av_), using root mean square (RMS) statistics, were generated. Using standard unit conversions, average P-wave interval velocities (v_av_) were calculated from Δt_av_.

The final dataset consists of twelve Log ASCII Standard (LAS v2.0) files, one for each exploration well. Each file includes metadata and curves of original transit time, averaged transit time, and calculated average P-wave velocity versus depth. Moreover, outputs in PDF format of the lithostratigraphic framework and associated log data were generated for each exploration well.

The dataset can be reused for seismic velocity modelling, time–depth conversion, basin and structural analysis, and subsurface interpretation in offshore settings. The standardized format allows for a direct integration into a wide range of subsurface software and facilitates the comparison with other regional or basin-scale velocity datasets. In addition, the dataset provides a reproducible digital reference for lithostratigraphic velocity information derived from legacy exploration wells in the Adriatic Sea.

Specifications Table

**Table:** 

Subject	Earth & Environmental Sciences
Specific subject area	Geology
Type of data	Log ASCII Standard (LAS) files
Data collection	Sonic log data were collected from legacy offshore exploration wells using raster well-log documents retrieved from the public ViDEPI database. Interval transit time (Δt) and depth values were obtained from previously digitized datasets and processed using Petrel™ software. Lithostratigraphic units were identified from raster stratigraphic logs and used to segment the sonic logs. Average transit times were calculated by block averaging and RMS statistics, and P-wave velocities were derived through unit conversion. Data were exported in LAS v2.0 format.
Data source location	Data of exploration wells from the middle/southern Adriatic Sea reported as raster documents within the ViDEPI project, at www.videpi.com, and CSV files in the Mendeley Data repository, at https://data.mendeley.com/datasets/xncfj4mrmp
Data accessibility	Repository name: Mendeley Data Data identification number: 10.17632/xrb2ny6scm Direct URL to data: https://data.mendeley.com/datasets/xrb2ny6scm
Related research article	M. Cicala, V. Festa, A. Marrone, Data extraction from vintage well sonic log graphs in the ViDEPI project (offshore the Apulia, southern Italy): a multi-useful dataset, Data Br. 46 (2023) 108,814, doi: 10.1016/j.dib.2022.108814

## 1 Value of the Data

• The dataset provides machine-readable Log ASCII Standard files derived from legacy offshore exploration wells. It should be noted that, the used data consisted in previously converted legacy analog well-log information into digital formats for their reuse. In this respect, original raster well-log documents from the public Visibility of Data related to Oil Exploration Activities in Italy (ViDEPI) database were digitized to obtain sonic log data in CSV format, and consisted of interval transit time (Δt) values and their corresponding depths.
• These digital data enable reuse by other researchers for modern geological/geophysical workflows, i.e., for time–depth conversion, seismic velocity modelling, and calibration of seismic interpretation, as the dataset includes average P-wave interval velocities (v_av_) constrained by lithostratigraphic units. By the use of Petrel™, the values of v_av_ were calculated from average interval transit times (Δt_av_), which in turn were obtained from Δt.
• The dataset is valuable as it applies a uniform processing workflow to all the considered wells, ensuring internal consistency across velocity estimates and allowing comparison with other regional or basin-scale datasets. Moreover, the processing workflow is valuable for those comparable situations, everywhere, characterized by original lithostratigraphic and sonic logs as analogic, raster images.
• The choice of Log ASCII Standard files as output data, is due to their reusability within open-source and commercial subsurface software to support seismic processing, basin and structural modelling, as well as educational applications, hence, without the need for re-digitization or format conversion.
• The data are useful for regional benchmarking, as they provide updated ranges of v_av_ for lithostratigraphic units in the middle and southern Adriatic Sea, that can be compared with existing P-wave interval velocities. Finally, this dataset represents the unique digital compilation in the study area that can be used as reference inputs in future studies.

## 2 Background

**Fig. 1:**
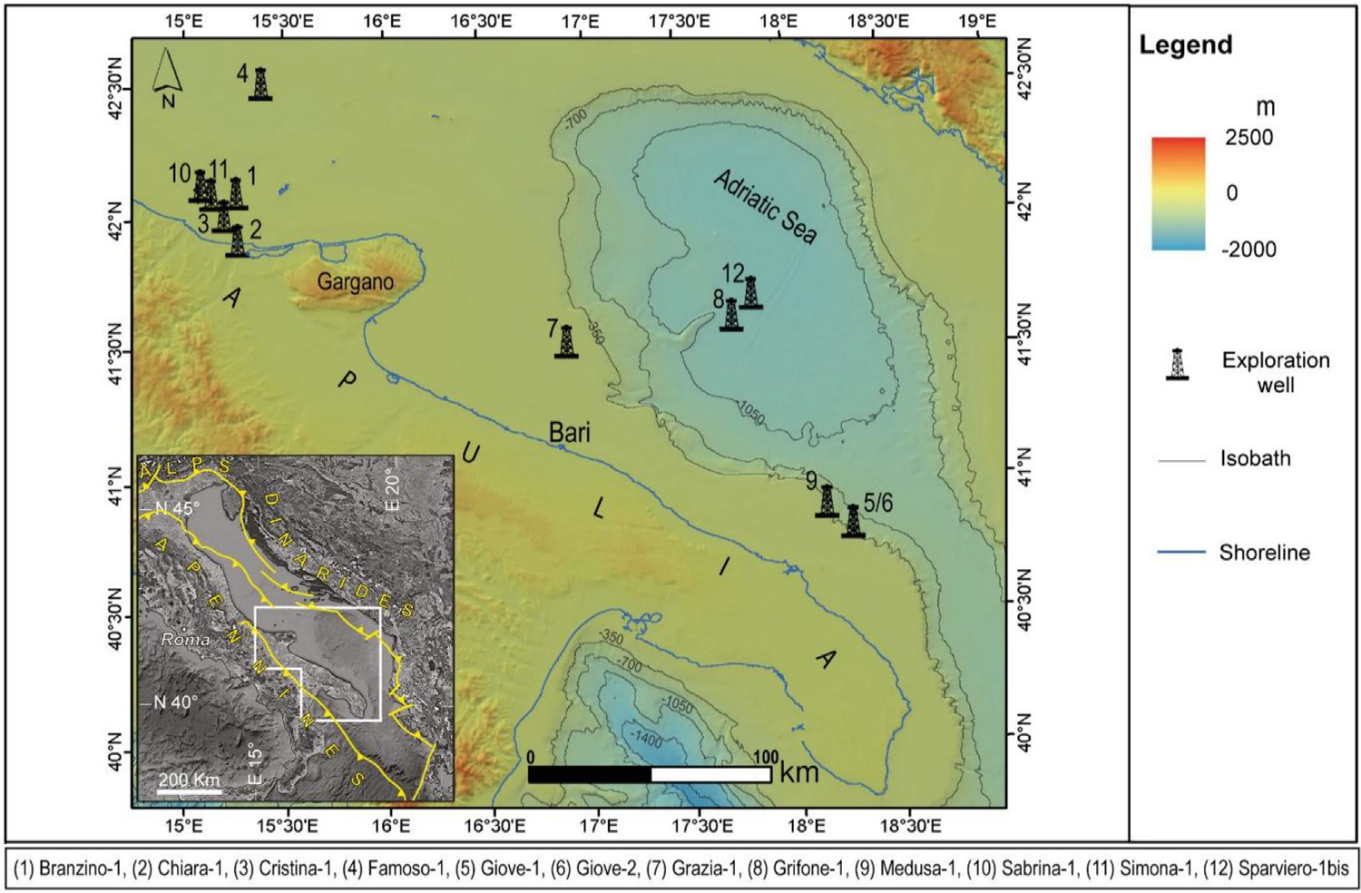
Topographic map along the middle and southern Adriatic Sea, with the location and names of the exploration wells included in this data paper. These wells feature raster sonic logs, with digitalized peaks tabulated in CSV files [1]. The white box indicates the study area. DTM data available from the GEBCO Digital Bathymetry [2] and processed with ArcGIS Pro software (Esri inc., version 3.5, 2025).

The compilation of this dataset was motivated by the need to make legacy offshore well-log information available in a standardized and reusable digital format. In the middle and southern Adriatic Sea (Southeastern Italy), lithostratigraphic and sonic log data from twelve exploration wells (Fig. 1) are commonly accessible only as historical raster images, which currently limits their direct use in quantitative geophysical workflows. These data, as original poor-quality raster images, are available by the ViDEPI project (www.videpi.com), i.e., *Visibilità dei Dati afferenti all'attività di Esplorazione Petrolifera in Italia*—Visibility of Data related to Oil Exploration Activities in Italy, whose data are freely accessible since 2007. From a methodological perspective, sonic logs provide fundamental constraints on seismic velocity models and time–depth relationships, but their effective reuse requires consistent digital processing and lithostratigraphic referencing. Within this context, Cicala et al. [1] laid the groundwork by extracting sonic data from raster images (interval transit time vs depth) and converting them into publicly available tabulated CSV files. Building on that previous work and datasets [1], the main objective of this paper is to construct ASCII metadata of each exploration well, including lithostratigraphic relationships/correlation, average interval transit time and average P-wave velocities logs.

## 3 BRIEF Geodynamic AND Paleoenvironmental Setting

**Fig. 2:**
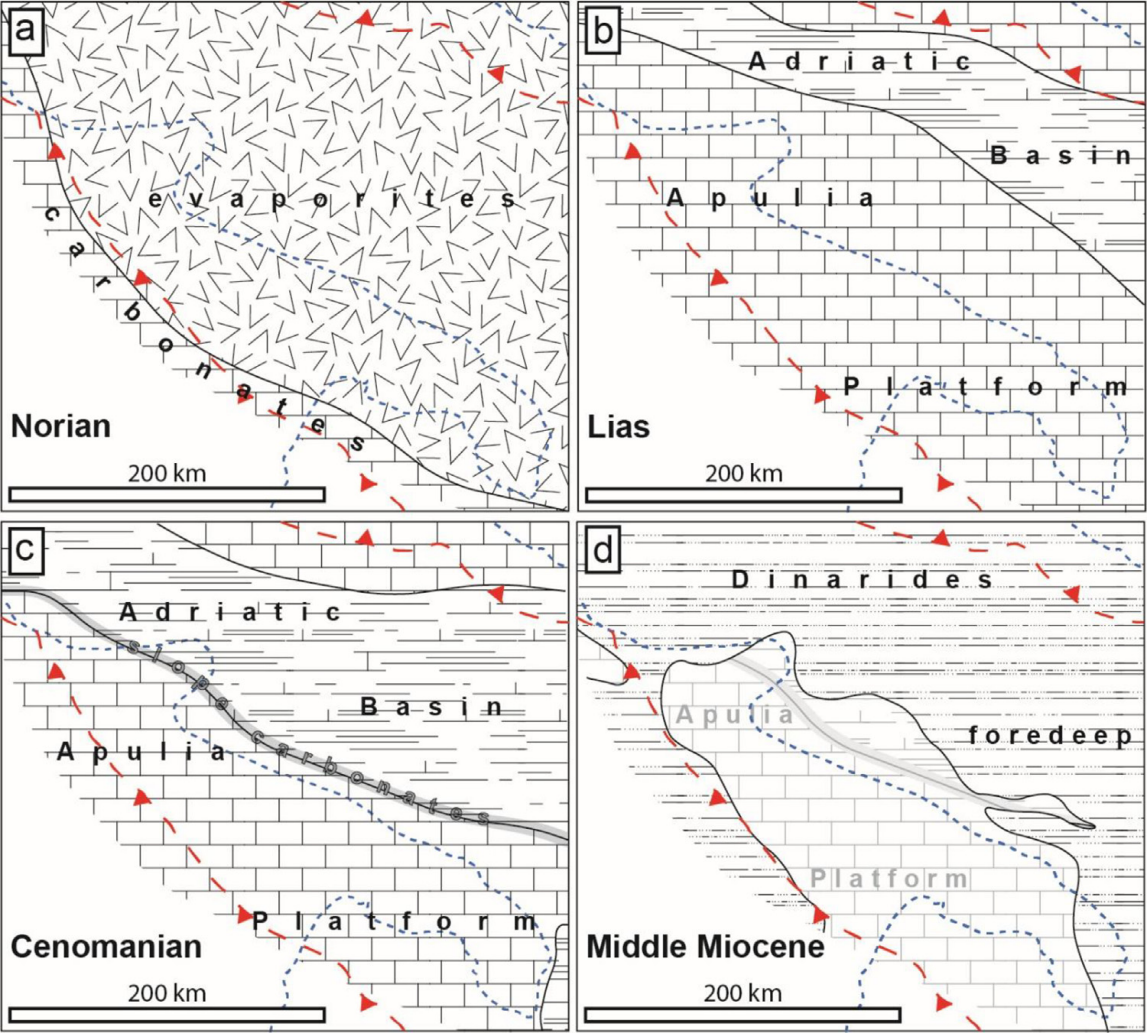
Paleogeographic sketches for the area of Apulia region and surroundings (the area corresponds to that of Fig. 1). The coastal line and the front of Apennines (southwest) and Dinarides (northeast) are also traced with blue and red dashed lines, respectively. a) Noric frame, modified after [5] and [6]. b) Lias frame, modified after [13]. c) Cenomanian frame, modified after [4], and references therein. d) Middle Miocene frame, after [8] and [14]; note the exposed, transparent areas dominated by the oldest carbonates.

Geological and geophysical investigations carried out in the Apulian region, and surroundings (Fig. 1) indicate that its paleogeographic evolution is closely related to the major deformational events that have affected the southern sector of the African Promontory (a sort of northward protuberance of the African plate), since the Late Paleozoic [3]. The principal geodynamic phases and related paleoenvironmental implications can be summarized as follows. An initial rifting stage of Pangea led to the development of a passive continental margin, with the African Promontory acting as such from the Late Paleozoic, in which the formation of continental basins dominated by red beds deposition occurred [3]. The onset of the separation of the Apulian Plate from the African Promontory happen during Triassic ([4], and references therein), with the development of a wide intra-continental (epeiric) shallow-water carbonate platform, in which marine evaporites deposited together with lagoon to shelves carbonates [5,6] (Fig. 2a). From Lower Jurassic the evolution of the Apulia Platform and the adjacent Adriatic Basin, i.e., the Umbria-Marche Basin, protracted within this rifting setting [3,7], with sedimentation in carbonate platform–carbonate ramp/slope–pelagic basin lateral association of paleoenvironments (Fig. 2b,c) [[8], [9], [10]]. According to [11], this geodynamic/stratigraphic evolution extended up to Eocene, when the Apulia Platform–Adriatic Basin system and overlying Paleocene carbonates, was involved in the Dinarides orogeny (to the east). Therefore, at least from early Oligocene the Adriatic Basin evolved as foredeep basin where pelagic sedimentation continued; the related proximal carbonate ramp deposits occurred at the outer margin of the foredeep, whose subsidence partially involved the Apulia Platform as well [11] (Fig. 2d). Moreover, late Miocene – Quaternary deposition occurred in the Dinarides foredeep [9] (e.g. Fig 2d); in the meantime, north of the Gargano promontory, the sedimentation happened in the Apennines foredeep domain during the involvement of the Apulia Platform–Adriatic Basin system in the tectono-sedimentary evolution of the Apennines (to the west) (e.g. Fig. 2d) [10,12].

## 4 Data Description

Twelve Log ASCII Standard (LAS) files have been obtained for each exploration well (Fig. 1 for their location), through export from Petrel™ (version 2024.9.0, Schlumberger, 2025). The LAS format is a standardized ASCII structure designed for well log data exchange. According to the LAS Version 2.0 specification [15], the metadata previously entered in Petrel™ are structured in the following four mandatory sections: “Version information”, “Well”, “Curve” and “Ascii”.

The “Version information” section refers to the used LAS version, which is 2.0 for all the twelve analyzed exploration wells.

The “Well” section includes the following indications/lines: “Top Depth”, “Bottom Depth”, “Increment”, “Null Value”, “Company”, “Well Name”, “Location”, “Location Type”, “Log Export Date”. “Top Depth” and “Bottom Depth” correspond to the depths automatically imported from the first and last depth values, respectively, already present in the .TXT input file (converted from the CSV file by [1]). In other words, they correspond to the depths of the top and bottom of the sonic log, respectively, recorded in the exploration well; as specified in Cicala et al. [1], these values take into account both the sea water column and the height of the rotary table. “Increment” value indicates the step size (i.e., spacing between depth points in the sonic log curve); the value of 0 is automatically assigned by Petrel™, because of not constant step size. As regards “Null Value”, which considers eventual lack of data, the default value −999.25 is indicated according to Petrel™; however, no null values exist in the input files. “Company” refers to the name of the company that owns the exploration well. Needless to say, “Well Name” is the proper name of the exploration well. “Location” includes the geographic coordinates (latitude and longitude, referenced to the WGS84 geodetic datum) of the exploration well. “Location Type”, is added here as a custom attribute and is “offshore” for all the exploration wells. “Log Export Date” is the year of the original well logging operations during drilling.

The “Curve” section is preparatory to the subsequent “Ascii” section, and it specifies the meaning of the following parameters: depth [m] (m = meters); interval transit time, Δt [µs/ft] (µs = microseconds, ft = feet); average interval transit time, Δt_av_ [µs/ft]; P-waves average velocity, v_av_ [m/s] (s = seconds).

Finally, the “Ascii” section contains the table of structured data values, from left to right, in the following columns: depth, v_av_, Δt and Δt_av_.

Therefore, the resulting twelve LAS files, one for each exploration well, are: Branzino-1.las, Chiara-1.las, Cristina-1.las, Famoso-1.las, Giove-1.las, Giove-2.las, Grazia-1.las, Grifone-1.las, Medusa-1.las, Sabrina-1.las, Simona-1.las, and Sparviero-1bis.las.

## 5 Experimental Design, Materials and Methods

**Fig. 3:**
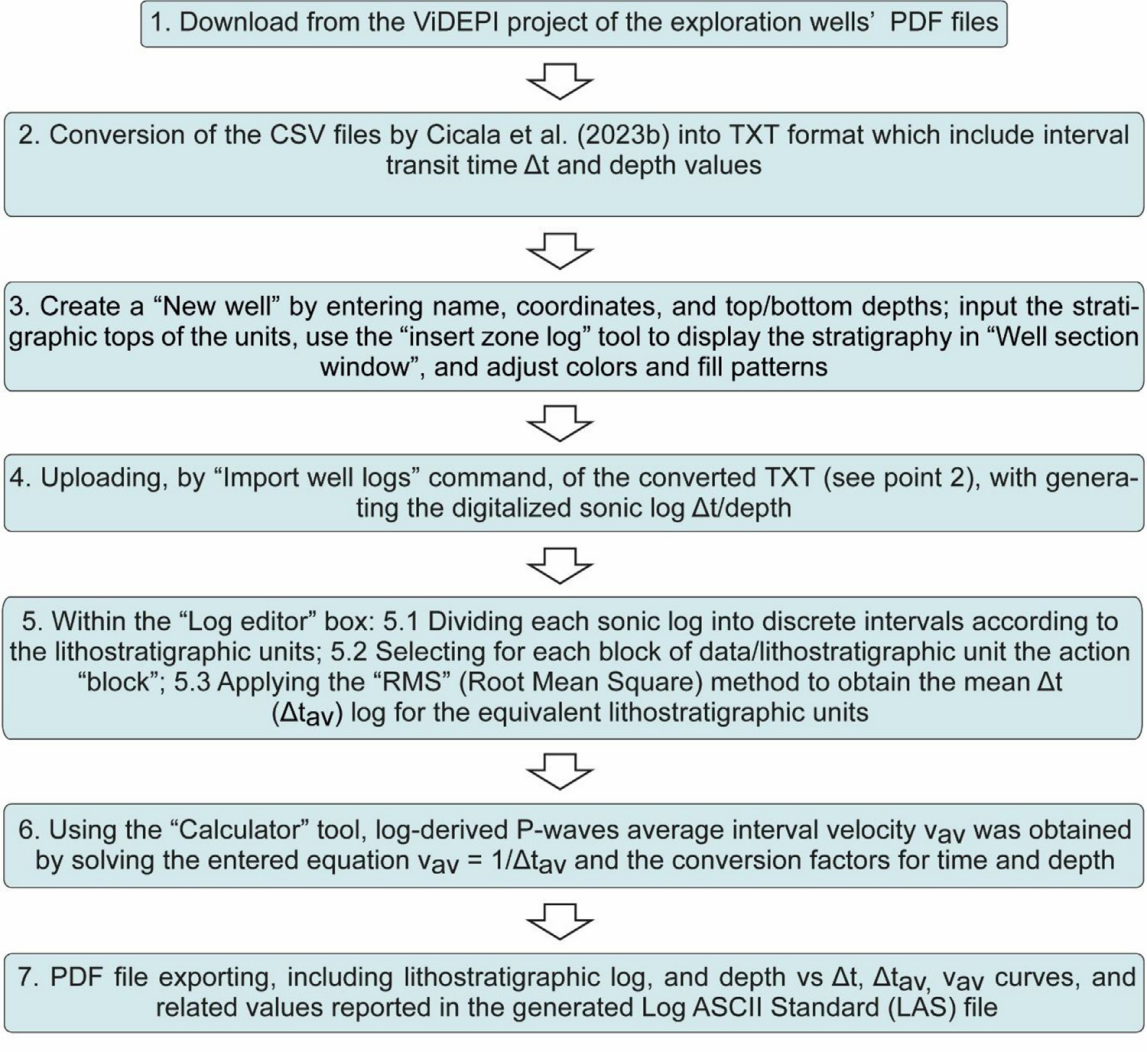
Data processing workflow for each exploration well data; stages 3 – 7 were performed using the software Petrel™.

To assess the average P-wave velocities of lithostratigraphic units in the middle and southern Adriatic Sea, the data were derived from the above mentioned twelve exploration wells (location in Fig. 1). As reported in the first step of the workflow of Fig. 3, the original PDF files of the raster exploration well logs were downloaded from the public database ViDEPI project; these documents provided the stratigraphic logs, which represent fundamental data (although as raster images) for the present analysis. So, the following links have been used to download the original PDF files for the corresponding Branzino-1, Chiara-1, Cristina-1, Famoso-1, Giove-1, Giove-2, Grazia-1, Grifone-1, Medusa-1, Sabrina-1, Simona-1, and Sparviero-1bis exploration wells: https://www.videpi.com/deposito/pozzi/profili/pdf/branzino_001.pdf, https://www.videpi.com/deposito/pozzi/profili/pdf/chiara_001.pdf, https://www.videpi.com/deposito/pozzi/profili/pdf/cristina_001.pdf, https://www.videpi.com/deposito/pozzi/profili/pdf/famoso_001.pdf, https://www.videpi.com/deposito/pozzi/profili/pdf/giove_001.pdf, https://www.videpi.com/deposito/pozzi/profili/pdf/giove_002.pdf, https://www.videpi.com/deposito/pozzi/profili/pdf/grazia_001.pdf, https://www.videpi.com/deposito/pozzi/profili/pdf/grifone_001.pdf, https://www.videpi.com/deposito/pozzi/profili/pdf/medusa_001.pdf, https://www.videpi.com/deposito/pozzi/profili/pdf/sabrina_001.pdf, https://www.videpi.com/deposito/pozzi/profili/pdf/simona_001.pdf.

The digitalized sonic log data from the twelve exploration wells, originally included in the raster PDF files, are available in CSV format within the database built by Cicala et al. [1]: each file is structured in two columns, with Δt [µs/ft] in column A and depth [m] in column B. Data processing and analysis were performed using the software Petrel™. To enable the use of the data within Petrel™ software, the original CSV files containing Δt and depth values, were converted into TXT format (point 2 in Fig. 3).

**Fig. 4:**
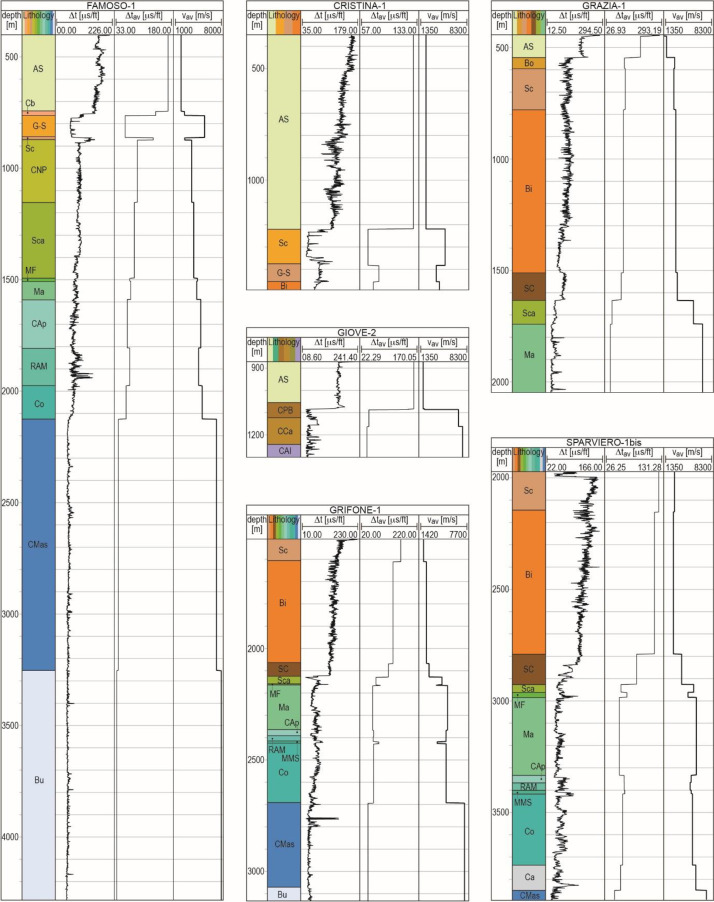

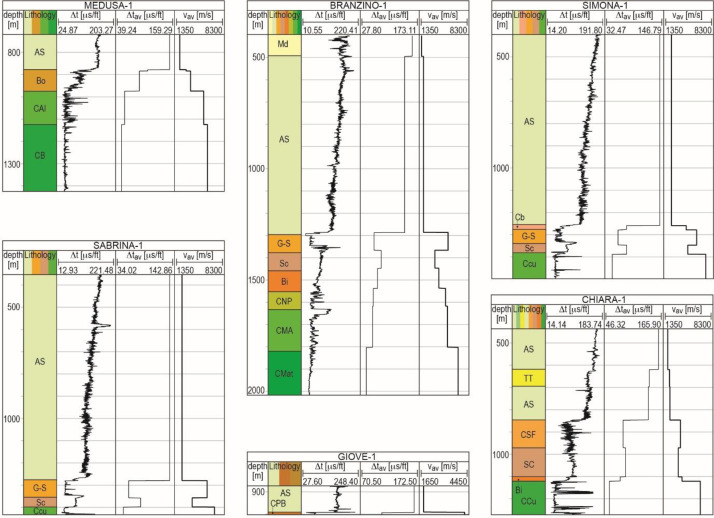
Lithostratigraphic, Δt, Δt_av_ and v_av_ logs for each exploration well (see Fig. 1 for their location) exported by Petrel™ as PDF files, and slightly modified with Adobe Illustrator (Adobe Inc., version 29.8.2) for editing purposes; acronyms of lithostratigraphic units are provided in Table 1.

The estimation of the average seismic P-wave velocities for the lithostratigraphic units followed a multi-step procedure with Petrel™, as summarized from step 3 to step 7 in the workflow of Fig. 3. These operations were applied for all twelve exploration wells considered in the present study. Each lithostratigraphic unit was identified from the raster lithostratigraphic log. A “New well” was created in the software by entering the name, coordinates and top and bottom depths of the exploration well (point 3 in Fig. 3). The lithostratigraphic succession was then constructed by manually entering, in the “well tops spreadsheet”, the depth values (“MD”, i.e., measure depth, attribute) for the top of each lithostratigraphic unit. To display the stratigraphy of the exploration well in the “Well Section window”, the entered tops were used to generate a lithostratigraphic log through “insert zone log” tool, and the corresponding unit acronym were assigned (see Table 1). The visual appearance of the resulting log, including colors and fill patterns, was then adjusted in the “Template settings” (point 3 in Fig. 3). It should be noted that the original recording depths of the sonic log implies the thickness of the equivalent lithostratigraphic log, as shown in Fig. 4. Subsequently, we used the “Import well logs” command into the converted TXT (see point 2 in Fig. 3), and the digitalized sonic log Δt/depth was generated for each exploration well (point 4 in Fig. 3, Fig. 4). Using the “block” action within the “Log editor” box, each sonic log was divided into discrete intervals according to the lithostratigraphic units (point 5.1 in Fig. 3). As a matter of fact, a significant variation of Δt can be observed between subsequent lithostratigraphic units. However, some Δt peaks are present within the same lithostratigraphic unit due to different lithotypes, porosity and/or fluid concentration within interval levels (Fig. 4). As described by [16], the “block” action allows bed-thickness correction, reduction of random errors, and consistent segmentation of the log. Hence, this action was useful for the reduction of Δt peaks within each block of data/lithostratigraphic unit (point 5.2 in Fig. 3). With the same “Log editor” box, we applied the statistical method of Root Mean Square (RMS) to the Δt values which calculated a representative weighted average (mean Δt, i.e., Δt_av_) for the equivalent lithostratigraphic units within the same exploration well (point 5.3 in Fig. 3, Fig. 4). The RMS values enable the identification of significant variations in the properties of drilled lithostratigraphic units. With the “Calculator” tool, log-derived P-waves average interval velocity (v_av_ [m/s]) values were obtained by solving the equation v_av_ = 1/Δt_av_, with a conversion factor of 1 µs = 10⁻⁶ s and 1 ft = 0.3048 m. Finally, the v_av_/depth for the equivalent lithostratigraphic units of the exploration well was generated (point 6 in Fig. 3, Fig. 4). Lastly, data were exported in LAS format file (point 7 in Fig. 3) (see the previous Data Description section for the details). Processed data were also exported by Petrel™ as PDF files for each exploration well, including lithostratigraphic, Δt, Δt_av_ and v_av_ logs (Fig. 4).

**Table 1:** Range of P-wave average velocity determined for the Triassic – Quaternary lithostratigraphic units.

Lithostratigraphic unit	Acronym	Dominant lithotype	Paleoenvironmental/geodynamic domain	P-wave average velocity
(Age)				[m/s]
Argille del Santerno	AS	clay	pelagic/Apennines-Dinarides foredeeps	1655 - 2485
(Lower Pliocene - Pleistocene)				
Montedoro	Md	sand and clay	pelagic/Apennines foredeeps	1930
(Pleistocene)				
Torrente Tona	TT	interbedded quartz sand and clay	pelagic/Apennines foredeeps	2220
(Upper Pliocene)				
Colombacci	Cb	clay-rich limestone and marl	continental-transitional/Apennines foredeep	2395
(Messinian)				
Gessoso-Solfifera	G-S	gypsum and chalk	marine evaporitic/Apeninnines-Dinarides foredeeps	5035 - 5890
(Messinian)				
Calcari di San Ferdinando	CSF	limestone	continental-transitional/Apennines foredeep	3730
(Tortonian - Messinian)				
Schlier	*Sc*	limestone with clay-rich marl	pelagic/Apennines-Dinarides foredeeps	1945 - 4580
(Serravalian - Turonian)				
Bisciaro	Bi	clay-rich limestone	pelagic/Apennines-Dinarides foredeeps	2325 - 4285
(Lower Miocene)				
Scaglia Cinerea	SC	marl with intercalation of limestone	pelagic/Apennines-Dinarides foredeeps	2665 - 3850
(Upper Oligocene - Middle Miocene)				
Calcari a Nummuliti di Peschici	CNP	clay-rich limestone	carbonate ramp/Adria passive margin-Dinarides foredeep	3450 - 5060
(Upper Eocene - Middle Oligocene)				
Bolognano	Bo	limestone and calcarenite	carbonate ramp/Apennines-Dinarides foredeeps	2780 - 3575
(Eocene?-Miocene)				
Calcareniti di Porto Badisco	Cca	bioclastic limestone	carbonate ramp/Adria passive margin-Dinarides foredeep	5195
(Upper Oligocene)				
Calcari di Castro	CPB	bioclastic limestone	carbonate ramp/Adria passive margin-Dinarides foredeep	7685
(Lower Oligocene)				
Scaglia	Sca	limestone with nodules and layers of chert	pelagic basin/Adria passive margin	3775 - 5545
(Upper Cretaceous - Middle Eocene)				
Calcari di Mattinata	CMat	limestone	carbonate slope/Adria passive margin	6690
(Upper Cretaceous)				
Calcari di Monte Acuto	CMA	limestone	carbonate slope/Adria passive margin	5290
(Upper Cretaceous)				
Calcare di Altamura	CAl	fossiliferous limestone	carbonate platform/Adria passive margin	5420
(Upper Cretaceous)				
Calcare di Bari	CB	fossiliferous limestone	carbonate platform/Adria passive margin	5945
(Lower Cretaceous)				
Calcari di Cupello	CCu	fossiliferous limestone	carbonate platform/Adria passive margin	6500 - 7105
(Lower Cretaceous)				
Marne a Fucoidi	MF	clay-rich limestone	pelagic basin/Adria passive margin	3550 - 4745
(Aptian - Albian)				
Maiolica	Ma	limestone	pelagic basin/Adria passive margin	4205 - 6650
(Tithonian - Barremian)				
Calcari ad Aptici	CAp	fossiliferous limestone	pelagic basin/Adria passive margin	4810 - 5195
(Dogger-Malm)				
Rosso Ammonitico Marchigiano	RAM	fossiliferous limestone	pelagic basin/Adria passive margin	4500 - 4830
(Lias)				
Marne di Monte Serrone	MMS	marl	pelagic basin/Adria passive margin	3700 - 5205
(Lias)				
Corniola	Co	limestone	pelagic basin/Adria passive margin	4845 - 5365
(Lias)				
Calcari anossici	Ca	radiolarian limestone	carbonate platform/Adria passive margin	5835
(Lias)				
Calcare Massiccio	CMas	limestone	carbonate platform/Adria passive margin	7145 - 7315
(Lias)				
Burano	Bu	dolostone and anhydrite	intra-continental shallow marine water basin to restricted carbonate platform/Pangea rift	7180 - 7850
(Trias)

In addition to the main product of the present paper (i.e., the LAS dataset for the considered exploration wells), this work also provides new reference ranges of v_av_ for the lithostratigraphic units in the middle and southern Adriatic Sea, which is a novel information useful for future studies along the region. In that sense, the new velocity values represent an upgrade/update for the regional seismic interval velocities previously provided by Bally et al. [17], an important reference for regional studies. Table 1 shows the new v_av_ values for the same lithostratigraphic intervals of *Figure 12* of Bally et al. [17]. In Table 1, ages and dominant lithotypes as in the lithostratigraphic log of analyzed exploration wells from ViDEPI; paleoenvironmental/geodynamic domain as in literature [6,[8], [9], [10], [11], [12],[18], [19], [20]].

## Limitations and Precautions

The exploration wells are distributed across a broad region of the middle and southern Adriatic Sea and intersect the same lithostratigraphic units at varying depths, each with distinct petrophysical properties. As a result, the average P-wave velocity values for these units (see Table 1) represent reference ranges rather than fixed values. When using this database, it is recommended that users select the average P-wave velocity (vav) from the LAS file corresponding to the exploration well(s) most relevant to their area of interest.

It should be considered that, according to the tectono-stratigraphic evolution by research studies regarding the middle and southern Adriatic Sea [[8], [9], [10],[18], [19], [20]] the lithostratigraphic units drilled in the exploration wells belong to the paleoenvironmental/geodynamic domain as reported in Table 1. More in detail, explicative and comprehensive 2D sketches of the stratigraphic architecture regarding the analyzed lithostratigraphic units (Table 1) are shown in: Fig. 4 of [9], *Figure 18* of [10] and *Figure 13* of [6].

## Ethics Statement

This work does not involve the use of human subject, does not involve animal experiment and does not involve data collected from social media platforms.

## CRediT Author Statement

**Christina Lombardo:** Data Processing, Writing - Original Draft and Validation; **Marianna Cicala:** Data Processing, Writing - Original Draft, Validation, Writing - Review & Editing; **Francesco De Giosa:** Methodology, Validation; **Luisa Sabato:** Writing - Review & Editing; **Giovanni Scicchitano:** Writing - Review & Editing; **Luigi Spalluto:** Writing - Review & Editing; **Gabriel Tagliaro:** Writing - Original Draft, Writing - Review & Editing; **Marcello Tropeano:** Writing - Review & Editing; **Vincenzo Festa:** Conceptualization, Writing - Original Draft, Writing - Review & Editing, Supervision.

## Data Availability

Mendeley DataSonic Log LAS Files from offshore exploration wells in the middle and southern Adriatic Sea (Italy), derived from the ViDEPI Project (Original data).
